# Digitization of rehabilitation evaluation and activity monitoring using thermography: protocol for a mixed-methods randomized multiple baseline study in inpatient rehabilitation

**DOI:** 10.3389/fdgth.2026.1884320

**Published:** 2026-08-18

**Authors:** Ravi Shankar, Karen Chua, Rathi Ratha Krishnan, Shuen-Loong Tham, Llewelyn Yi Chang Tan, Aletheia Lai, Megan Si En Lau, Zhi Kai Koh, Ernest Thia, Yating Su, Srinikethan Madapuzi Srinivasan, Poo Lee Ong

**Affiliations:** 1 Clinical Research & Innovation Office, Tan Tock Seng Hospital, Singapore, Singapore; 2 Rehabilitation Research Institute of Singapore (RRIS), Nanyang Technological University, Singapore, Singapore; 3 Institute of Rehabilitation Excellence (IREx), Tan Tock Seng Hospital Rehabilitation Centre, Singapore, Singapore; 4 Nursing Division, Tan Tock Seng Hospital, Singapore, Singapore; 5 CoNEX Healthcare Pte Ltd, Singapore, Singapore

**Keywords:** activity monitoring, artificial intelligence, digital health, inpatient rehabilitation, mixed methods, thermography

## Abstract

**Background:**

Patients in inpatient rehabilitation spend significant time of their day in sedentary activities, limiting recovery potential. Current monitoring relies on manual, intermittent nursing observations that are labor-intensive and produce fragmented data. Thermography-based artificial intelligence (AI) monitoring offers a privacy-preserving, continuous alternative; however, its impact on rehabilitation engagement when combined with patient-facing feedback has not been rigorously evaluated. Conventional pre-post and parallel-group designs are poorly suited for ward-level digital health interventions because of contamination risks, ethical constraints on withholding technology, and failure to exploit dense within-patient time-series data.

**Objective:**

This study aims to evaluate the effectiveness of the PreSAGE thermography-based AI activity monitoring and feedback dashboard system (the DREAMT intervention) in enhancing patient physical activity levels, rehabilitation outcomes, and nursing workflow, and to explore the experiences and contextual factors influencing uptake.

**Methods:**

This is an explanatory sequential mixed-methods study embedding a randomized multiple baseline design across participants (RMBD) for the quantitative component and semi-structured interviews for the qualitative component. Conducted over 24 months at Tan Tock Seng Hospital, Singapore, the study proceeds through three phases: (1) AI algorithm development via thermographic data collection and manual labelling (*n* = 50); (2) algorithm validation and user-centered dashboard co-design; and (3) RMBD evaluation (*n* = 50), in which each patient undergoes continuous monitoring from admission and the feedback dashboard is activated at a randomly assigned day (3–7 post-admission), with each patient serving as their own control. The primary outcome is daily total active time. Secondary outcomes include exercise frequency, Functional Independence Measure scores, length of stay, nursing workload, and dashboard utilization. Quantitative data will be analyzed using piecewise multilevel growth models. Qualitative interviews with 10–15 patients, 2–3 ward sisters, and 4–6 clinicians will be analyzed using reflexive thematic analysis, with integration via a joint display framework.

**Conclusions:**

This protocol outlines a methodologically rigorous evaluation of a novel privacy-preserving AI monitoring and feedback system. The RMBD utilizes continuous within-patient data to yield stronger causal inference than conventional designs while remaining feasible in a single-site setting. If effective, DREAMT could shift rehabilitation monitoring from episodic, subjective observation to continuous, personalized, data-driven care.

## Introduction

### Background

Prolonged physical inactivity during hospitalization poses substantial risks to patient health and recovery. Hospitalized adults spend approximately 87% of their time in sedentary postures (1, 2), averaging fewer than 900 steps per day (3), even among patients who can ambulate independently (4). This inactivity accelerates muscle deconditioning, with measurable protein degradation occurring within 48 h of immobility (5), and is associated with adverse cognitive, functional, and quality-of-life outcomes (6, 7). The global economic burden of physical inactivity is estimated at US $54 billion in direct healthcare costs and 5.3 million deaths annually (8, 9).

In inpatient rehabilitation settings, where the explicit clinical goal is functional recovery, the consequences of inactivity are paradoxically acute. Patients typically engage in structured therapy for only 2 to 3 h per day (10–13), with the remaining waking hours largely unmonitored. Outside scheduled sessions, exercise compliance depends on intrinsic motivation and caregiver prompting, both of which are highly variable. Systematic tracking of patient activity is essential for identifying sedentary patterns, detecting exercise non-compliance, recognizing early signs of clinical deterioration or improvement, and enabling therapists to adjust treatment plans based on objective data rather than subjective impression (14).

Current monitoring practices rely on manual, intermittent observation and documentation by nursing staff, who must balance direct patient care with administrative duties. These visual checks produce fragmented, episodic records that lack the temporal resolution needed to characterize activity patterns over the course of a day or admission. The labour intensity of manual monitoring also diverts nursing resources from direct clinical care (15).

Several technological solutions have been explored. Wearable accelerometers (e.g., Fitbit, Garmin, EQ02 LifeMonitor) provide continuous data but require consistent patient compliance, regular charging, and connectivity management, which are challenging requirements for elderly or cognitively impaired rehabilitation populations (16). Optical (RGB) cameras enable detailed activity recognition but raise serious privacy concerns in clinical environments where patients may be undressed or receiving personal care (17). Light detection and ranging (LIDAR) systems (e.g., SoundEye Lasso) offer limited image quality and range, constraining detailed activity recognition (18, 19).

Thermography-based monitoring represents a promising alternative that addresses these limitations. Thermal imaging captures heat signatures rather than visual features, enabling person detection and posture recognition through AI models without recording personally identifiable information. The PreSAGE system, co-developed between Tan Tock Seng Hospital and CoNEX Healthcare Pte Ltd, is a patented inpatient monitoring solution commercially deployed across multiple healthcare institutions in Singapore for bed-exit prevention. This existing deployment provides the technological foundation for extending the system's capabilities of comprehensive activity monitoring and real-time feedback.

### Evidence for feedback-enhanced activity monitoring

Behavioural research consistently demonstrates that feedback is a powerful driver of health behaviour change. Kirwan et al. (20) showed that real-time feedback via smartphone significantly increased participants' physical activity. A meta-analysis by Laranjo et al. (21) found that digital feedback combined with personalized goal setting enhanced intervention effectiveness across diverse populations. A systematic review by Krukowski et al. (22) confirmed the importance of feedback in behavioural interventions while noting that optimal delivery methods and frequency require further investigation, particularly in clinical populations.

The PreSAGE system has undergone preliminary validation for patient activity classification, demonstrating the ability to automatically distinguish lying, sitting on bed edge, and sitting in a chair, and to recognize upper and lower limb exercise detection. These capabilities create the opportunity for a patient-facing feedback dashboard that translates continuous monitoring data into actionable information, thereby completing the feedback loop that current monitoring approaches lack.

### Study rationale and design selection

Selecting an appropriate research design for evaluating the DREAMT intervention requires careful consideration of the study context. Randomized controlled trials (RCTs) with parallel groups are challenging in this setting: randomizing patients within the same ward risks contamination (staff behaviour changes affect all patients), randomizing between wards requires more wards than are available, and withholding a potentially beneficial monitoring system from a control group raises ethical concerns (23). Simple pre-post designs, though commonly used in digital health evaluations, are vulnerable to maturation effects, secular trends, regression to the mean, and history threats, providing weak causal inference (24).

The controlled interrupted time series (CITS) design, used in many policy evaluations, requires a clearly defined population-level intervention point and is optimized for aggregate-level data with many time points before and after a single transition (25, 26). In the present study, the intervention operates at the individual patient level (each patient receives their own dashboard), and the continuously monitored data is naturally structured at the patient level rather than the ward level.

A randomized multiple baseline design across participants (RMBD) is better suited to this context (27–30). The multiple baseline design (MBD) is an established single-case experimental design (SCED) in rehabilitation research that involves: (1) continuous monitoring of each participant from enrolment (baseline A phase); (2) randomized assignment of the intervention onset point to different time points across participants, such that the intervention (B phase) is introduced in a staggered manner; and (3) comparison of pre-onset and post-onset trajectories within each participant, replicated across the sample (28, 31). In the MBD across participants, each individual begins in the baseline condition simultaneously, and the intervention is introduced sequentially to different participants at different, ideally randomized, time points (29, 32). If improvements systematically coincide with dashboard activation despite occurring at different calendar times and different days post-admission, the observed effects are unlikely to be attributable to maturation, secular trends, or history (28).

The randomization of intervention onset timing is a critical feature that enhances the internal validity of the MBD by controlling for potential biases in when the intervention is introduced (27, 28). This approach is consistent with recommendations by Kratochwill and Levin (28), who demonstrated that incorporating randomization into SCEDs substantially strengthens causal inference and enables the use of randomization-based statistical tests.

This design offers several advantages for the present study. First, it leverages the dense within-patient time-series data generated by continuous thermographic monitoring, which would be underutilized by between-group designs. Second, each patient serves as their own control, eliminating between-subject confounding. The substantial length of stay variability observed in Phase 1 (IQR 15–35 days) further supports a within-patient design, as this heterogeneity would introduce considerable noise in between-group comparisons. Third, the staggered onset across participants controls for time-related threats to validity without requiring a separate control ward. Fourth, the MBD is increasingly recognized in rehabilitation research as providing stronger, replication-dependent causal inference, relative to uncontrolled pre–post and single-group interrupted time-series designs, when replicated across a sufficient number of participants (27, 30, 33). Fifth, unlike CITS, the design naturally accommodates individual-level intervention delivery and variable patient trajectories. Sixth, the design meets the What Works Clearinghouse (WWC) standards for SCEDs when it includes at least three demonstration opportunities for the experimental effect at three different points in time (27), a criterion substantially exceeded in the present study with five onset strata and 50 participants.

Quantitative outcomes alone cannot explain why or how an intervention achieves its effects, or identify the contextual factors that facilitate or impede implementation. An explanatory sequential mixed-methods approach (34) embeds a qualitative process evaluation following the Medical Research Council framework for complex interventions (35), enabling exploration of patient and staff experiences, perceived barriers and facilitators, and the mechanisms through which dashboard feedback may influence behaviour.

### Objectives

The primary objective is to evaluate the effectiveness of the DREAMT intervention (PreSAGE thermography-based AI monitoring with activity feedback dashboard) in increasing daily patient physical activity levels during inpatient rehabilitation, using a randomized multiple baseline design across participants.

The secondary objectives are to: (1) assess the impact on clinical rehabilitation outcomes, including Functional Independence Measure (FIM) scores, Functional Ambulation Category (FAC) scores, Modified Barthel Index (MBI), length of stay, discharge destination, and complications; (2) evaluate the effect on nursing workload distribution and time allocation; (3) explore the experiences, perceptions, and recommendations of patients, nursing staff, and therapists through semi-structured interviews; and (4) integrate quantitative and qualitative findings to develop a comprehensive understanding of intervention effectiveness and implementation considerations.

## Methods

### Study design overview

This is an explanatory sequential mixed-methods study (34, 36) that embeds a randomized multiple baseline design across participants for the quantitative evaluation component and semi-structured interviews with reflexive thematic analysis for the qualitative component. The RMBD is a form of SCED in which the intervention is introduced in a staggered, randomized fashion across participants, with each participant serving as their own control (27–29). The quantitative phase provides evidence of intervention effects, and the subsequent qualitative phase helps explain and contextualize these effects. Integration occurs through a joint display framework (37). The study is reported in accordance with the SPIRIT 2025 statement (38) and the TREND statement (39) for transparent reporting of nonrandomized evaluation designs, supplemented by the SCRIBE 2016 guidelines (30) for reporting single-case experimental designs.

The study will be conducted across three sequential phases over 24 months at the Integrated Care Hub (ICH) of Tan Tock Seng Hospital, a tertiary rehabilitation facility in Singapore (Table 1).

**Table 1:** Timeline.

Phase	Duration	Key activities
Phase 1: AI Development	Months 1–6	PreSAGE data collection, patient recruitment (*n* = 50), manual activity labelling, AI model training and optimization
Phase 2: Validation & Dashboard	Months 7–12	Algorithm validation against clinician ground truth, iterative dashboard co-design, usability testing, interview guide development and piloting
Phase 3: RMBD Evaluation	Months 13–24	Patient recruitment (*n* = 50), randomized multiple baseline dashboard deployment, continuous data collection, qualitative interviews (months 20–24), data analysis, mixed-methods integration

### Study setting

The ICH is a dedicated inpatient rehabilitation facility within Tan Tock Seng Hospital providing multidisciplinary rehabilitation for patients recovering from stroke, traumatic brain injury, orthopedic conditions, and other disabling conditions. The ward comprises single-bed cubicles, each equipped with a ceiling-mounted PreSAGE thermography camera connected to a centralized processing platform. Patients receive therapy five days per week during admission, which may include physiotherapy, occupational therapy, speech therapy, dietician consultation and/or psychological services depending on individual needs, and each therapy session lasts up to one hour. Clinical documentation uses the EPIC electronic medical record system. Based on Phase 1 data (*n* = 33), the median rehabilitation length of stay is 24 days (mean 27.3, SD,5 17.0, IQR, 15–35, range 4–70 days).

### Phase 1: AI algorithm development and data collection (months 1–6)

#### Participants

Fifty patients admitted to the ICH will be recruited. Inclusion criteria are age 21 years or older, admission to the ICH within 4 weeks of acute admission, and a medically stable condition. Exclusion criteria are known intellectual disability or untreated psychiatric conditions that preclude informed consent, pregnancy or lactation, and inability to provide consent either directly or through a legally authorized representative.

#### Procedures

The PreSAGE system will continuously collect thermographic data at each patient's bedside throughout their inpatient stay. Research assistants will extract baseline clinical data from EPIC, including demographics, premorbid function, Clinical Frailty Scale score, diagnosis, and baseline functional assessments (FIM, FAC, MBI).

Trained research staff under clinician supervision will manually label patient activities captured by the thermographic system. A minimum of 3,000 labelled activity samples across 15 activity types will be generated, with at least 200 samples per activity type to capture within-activity variation. Activities include lying on bed (supine, left and right side-lying), sitting on bed edge, sitting in chair, standing, transferring (bed to chair and vice-versa), walking, eating, and exercise categories (upper limb and lower limb exercises). Nursing activities captured include assisted turning, assisted transfer, nursing patient engagement episodes and its duration.

Labelled data will train and refine AI activity recognition algorithms. The system architecture developed to date, together with its interim performance on the subset of data annotated so far, is summarized below.

The AI system employs a hierarchical two-stage architecture to classify posture, exercise activity, and behavioural engagement based on thermographic data generated from a ceiling-mounted position. The first stage uses a YOLOv8-based pose estimation model to detect human body key points from each thermographic frame. The model estimates 17 anatomical key points for each detected person and serves as the foundation for all downstream activity analysis. The pose estimation model was fine-tuned using 8,000 manually annotated thermographic images collected from 25 subjects (an interim subset of the Phase 1 development cohort). Images were partitioned into training, validation, and testing sets at the subject level to prevent data leakage arising from similar frames of the same subject appearing in multiple datasets. Data augmentation included random rotation, scaling, horizontal flipping, and brightness adjustment to improve robustness to variations in subject position and thermal appearance.

Pose estimation performance was evaluated using standard COCO key point detection metrics, including mean Average Precision (mAP) at IoU thresholds of 0.50 (AP50) and 0.50–0.95 (AP). The model achieved an AP50 of 92.6% and an AP of 60.0% for key point detection. Bounding box detection achieved an AP50 of 97.6% and an AP of 71.4%.

The second stage performs activity interpretation using the detected body keypoints. Patient posture classification is formulated as a supervised machine learning problem. Features derived from the detected keypoints include normalized x- and y-coordinates and confidence score. These features are used as input to a Random Forest classifier to categorize posture into four clinically relevant states: supine lying, left-side lying, right-side lying, and sitting on the bed edge. These four states constitute the bed-referenced posture classes; recognition of the additional out-of-bed activity states contributing to the primary activity-time outcome (sitting in a chair, standing, and walking) is being incorporated as the labelled dataset is expanded.

Random Forest (RF) hyperparameters, including the number of trees and maximum tree depth, were optimized using five-fold cross-validation on the training cohort. A minimum RF prediction probability threshold was applied to suppress uncertain classifications. Model performance was evaluated using accuracy (85.5%), precision (85.9%), recall (85.3%), and F1 score (85.5%). Confusion matrices were generated to identify commonly misclassified posture states.

Exercise detection is implemented using an algorithmic temporal movement analysis. Individual exercise repetitions are defined as a complete expansion-and-return movement cycle of specific body segments. A valid exercise episode is defined as eight or more consecutive and matched repetitions occurring within predefined temporal intervals. Thresholds governing movement amplitude, repetition duration, and continuity were determined through iterative analysis of manually labelled rehabilitation exercise sessions in consultation with clinical physiotherapists. The current algorithm supports detection of expansive upper- and lower-limb exercises performed on either the left or right side of the body.

Nursing activities are currently categorized into long-duration and short-duration interactions. To further distinguish different types of nursing activities, a temporal activity classification module is under development. This component employs a lightweight Temporal Convolutional Network (TCN) operating on sliding windows of pose sequences to learn temporal movement patterns associated with nursing assistance, patient transfers, and caregiver interactions. Performance of this module will be evaluated using accuracy, precision, recall, F1 score, and confusion matrix analysis following completion of algorithm development.

To ensure unbiased evaluation, all model development, hyperparameter optimization, and performance testing were conducted using patient-level rather than frame-level data partitioning, thereby preventing information leakage between training and evaluation datasets. Consequently, the reported performance metrics reflect the model's ability to generalize to previously unseen patients and behavioural patterns.

Classification errors were analysed through manual review of misclassified cases. Common failure modes included occlusion by nursing staff during assisted care, uncommon body configurations that were underrepresented in the training dataset, reduced visibility beneath blankets, and rehabilitation exercise postures that were absent from the training data. Ongoing model refinement focuses on expanding the training dataset with these challenging scenarios and incorporating temporal information to improve classification robustness.

### Phase 2: algorithm validation and dashboard co-design (months 7–12)

#### Algorithm validation

The labelled dataset will be partitioned at the patient level into training, validation, and testing sets to prevent information leakage between partitions. Model performance will be evaluated using accuracy, precision, recall, F1 score, area under the receiver operating characteristic curve, and confusion matrix analysis. Classification accuracy will be validated against clinician ground truth for 1,000 randomly selected activity samples from Phase 1 participants, stratified across morning, afternoon, and night periods in a 2:2:1 allocation ratio.

#### Dashboard co-design

An iterative, user-centred co-design process will develop the feedback dashboard in collaboration with clinicians, nurses, therapists, and patients. The technology partner (CoNEX Healthcare) will implement iterative prototypes based on design workshops. The dashboard will include patient activity summaries (daily active vs. sedentary time, sitting-out-of-bed episodes, exercise counts), trend visualizations across the admission, goal-setting features with progress tracking, sleep quality indicators, and nursing engagement metrics. Separate patient-facing (bedside tablet) and clinician-facing views will be developed. Usability testing will follow the System Usability Scale (40) with a target score above 68 (above average usability).

### Phase 3: randomized multiple baseline evaluation (months 13–24)

#### Study design

The primary quantitative evaluation employs a randomized multiple baseline design across participants (28, 29, 32), in which each patient undergoes two phases during their admission:

**Phase A (Baseline Observation):** From admission, the PreSAGE system continuously monitors patient activity. Activity data are collected and processed but no feedback is provided to the patient or treating team. This observation-only period provides individualized baseline data for each patient, lasting 3 to 7 days depending on the randomly assigned onset point (see Randomization below), consistent with the A phase in SCED methodology (27, 31).
**Phase B (Dashboard Intervention):** At the randomly assigned onset point, the patient-facing dashboard is activated on a bedside tablet and the clinician-facing dashboard becomes available to the treating team. Therapists set personalized activity goals, and patients receive continuous feedback on their progress. Thermographic monitoring continues throughout.

The critical design feature is the randomized timing of the A-to-B transition. Each patient will be randomly assigned, using a computer-generated allocation sequence with concealed envelopes, to one of five onset points: post-admission day 3, 4, 5, 6, or 7. If the assigned onset day falls on a weekend, activation will be deferred to the next weekday to ensure research assistant availability and clinical team presence for goal-setting. This ensures that each patient has a minimum baseline observation period of 72 h (to establish a stable pre-intervention trend) while staggering the transition point across the sample in a manner consistent with the MBD framework (29, 32). Ten patients will be assigned to each of the five onset strata, yielding 50 patients total.

**Figure 1:**
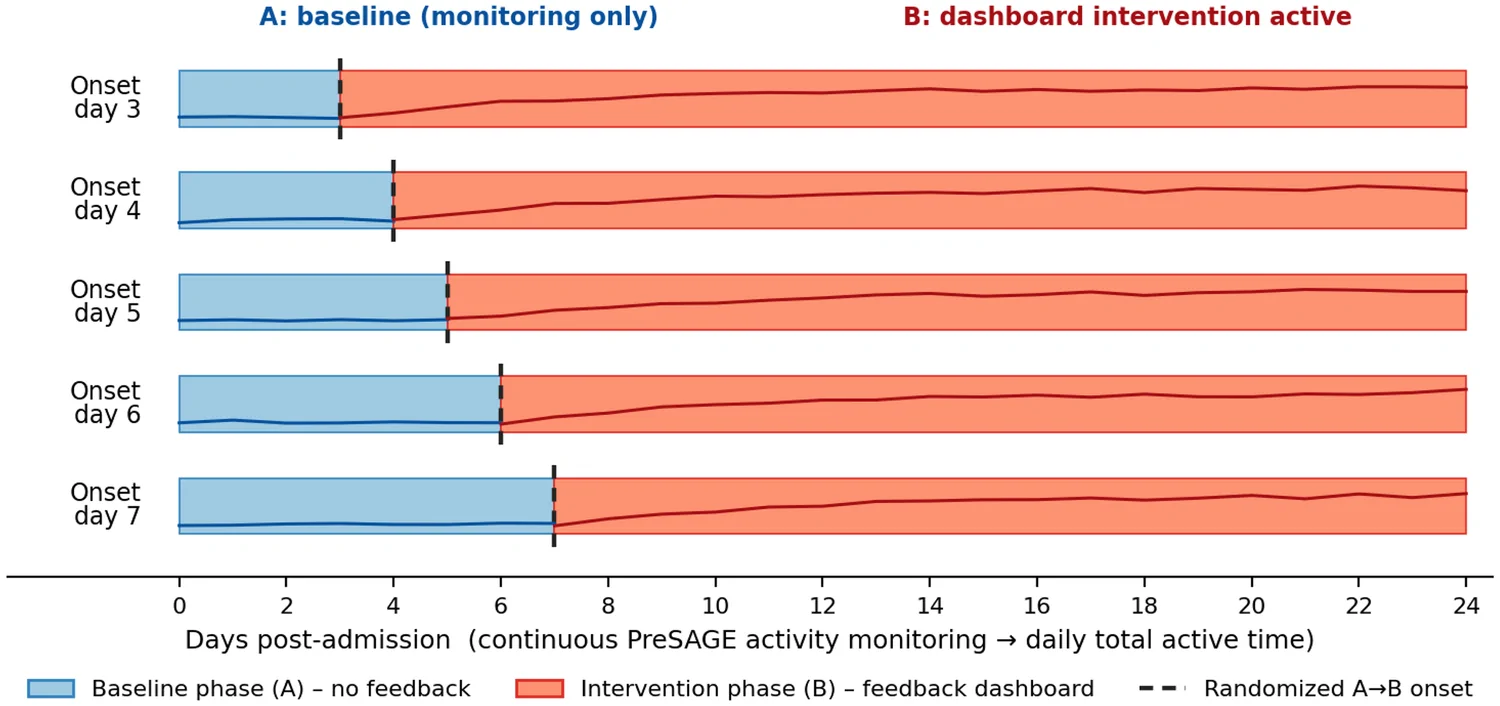
Schematic of the randomized multiple baseline design across participants. Each patient is continuously monitored from admission (baseline phase **A**, monitoring only) until a randomly assigned onset day (3–7 post-admission), at which the feedback dashboard is activated (intervention phase **B**). The staggered **A** → **B** transitions across the five onset strata, replicated across participants, allow improvements that coincide with dashboard activation to be distinguished from maturation, history, and regression to the mean. The daily total active time derived from continuous PreSAGE monitoring constitutes the primary outcome time series. Activity traces are illustrative.

If improvements in activity consistently emerge following dashboard activation, despite activation occurring at five different timepoints across participants, this provides strong evidence that the dashboard, rather than natural recovery or external factors, is responsible for the observed changes (28). The design effectively controls for maturation (natural recovery trajectory would not systematically align with randomly varied onset points), history (external events affect all patients regardless of onset assignment), and regression to the mean (pre-intervention trends are explicitly modelled). This logic of experimental control is identical to that of the concurrent MBD across participants: the demonstration of the experimental effect is replicated each time a new participant transitions from A to B at a different time point, while other participants remain in baseline (27, 31). With 50 participants across five strata, the study provides 50 such demonstrations, far exceeding the minimum of three required by SCED standards (27). A schematic of the design, showing the baseline phase, the staggered randomized A → B onset across the five strata, and the continuous outcome-measurement timeline, is provided in Figure 1.

### The DREAMT intervention

The DREAMT intervention is a multi-component digital health intervention comprising:

(1) *Continuous thermographic monitoring:* The PreSAGE system provides automated 24-hour activity data capture, processing, and classification using validated AI algorithms.
(2) *Patient-facing feedback dashboard:* For functionally independent patients, the dashboard is accessible via a bedside tablet, displaying daily activity summaries (active time, sitting episodes, exercise counts), graphical progress toward personalized goals, and motivational prompts. For functionally dependent patients who may be unable to independently access the tablet, the dashboard functions primarily as a clinician-facing tool, with the care team using activity data to guide nursing engagement, turning schedules, and early mobilization decisions.
(3) *Therapist-guided goal setting:* At the point of dashboard activation, therapists set initial personalized activity goals (e.g., daily minutes of sitting out of bed, exercise repetitions) based on the patient's baseline data and clinical status. Goals are reviewed and adjusted every 2–3 days using dashboard data.
(4) *Clinician-facing dashboard:* Providing the care team with activity trend data, nursing engagement metrics, sleep indicators, and patient progress visualizations to support clinical decision-making and care coordination.

### Outcome measures

The primary outcome is daily total active time (minutes per day), defined as time spent in non-lying postures (sitting on bed edge, sitting in chair, standing, walking, exercising) as continuously measured by the PreSAGE system. This outcome is aggregated daily and analysed as a within-patient time series. The validity of this AI-derived measure will be established against clinician-rated ground truth during Phase 2 validation (accuracy, precision, recall, F1, and AUROC), and only activity states meeting a pre-specified per-class reliability threshold (F1 ≥ 0.80) will contribute to the primary metric; states with lower reliability (for example, small-motion exercises) are reported descriptively and excluded from the primary outcome. Because individual rehabilitation prescriptions are heterogeneous and patient-specific, personalised therapist-set goals are evaluated as a patient-referenced secondary outcome rather than folded into the primary measure, which is defined identically for all participants to preserve comparability across the cohort.

Secondary quantitative outcomes include: (a) daily number and duration of sitting-out-of-bed episodes; (b) daily exercise frequency and duration, disaggregated by upper and lower limb exercises; (c) daily proportion of prescribed exercises completed (exercise compliance rate); (d) number of goals set and achieved per day; (e) dashboard utilization (daily interaction count and duration); (f) FIM score change from admission to discharge; (g) FAC score change; (h) MBI score change; (i) length of stay (days); (j) discharge destination (home, community hospital, skilled nursing facility); (k) incidence of complications (pressure injuries, deep vein thrombosis, falls, hospital-acquired infections); (l) nursing engagement frequency and duration across activity types (wound dressing, diaper change, dressing, intravenous management, patient calls, nasogastric tube feeding, assisted feeding, medication administration, vital signs monitoring), recorded as episode count and cumulative minutes per day; (m) time to first sit-out-of-bed episode post-admission; (n) complication rates (pressure injuries, deep vein thrombosis) analyzed by functional level subgroup.

### Sample size justification

Sample size estimation for the RMBD follows guidance from Kratochwill et al. (27) and Shadish et al. (41) on single-case experimental designs adapted for group-level analysis. The key considerations are: (a) the number of data points per phase per participant, and (b) the number of participants (replications).

With a median length of stay of 24 days (mean 27.3, SD 17.0) based on Phase 1 data, and randomized onset at days 3–7, each patient will contribute 3–7 baseline data points and a variable number of intervention data points. At the median stay, intervention points range from 17 to 21 days. Approximately 18% of Phase 1 patients had stays under 14 days; these patients will be included in the intention-to-treat analysis but contribute fewer post-onset observations. Shadish et al. (41) recommend a minimum of 3–5 data points per phase for SCED analyses. For the group-level multilevel analysis, we follow Moeyaert et al. (42), who demonstrated that 30 or more participants provide adequate power (>80%) for detecting medium effect sizes (d = 0.5) in multilevel models of SCED data with 20 time points per participant. Our planned sample of 50 patients with 21 daily data points each substantially exceeds this threshold.

Additionally, Monte Carlo power simulations (2,000 replications) were conducted to evaluate the study's ability to detect intervention effects under the piecewise multilevel growth model. Data were generated assuming 50 participants (10 per onset stratum), a median of 24 daily observations each (based on Phase 1 empirical length of stay; IQR 15–35 days), a within-person residual standard deviation of 25 min, between-patient intercept standard deviation of 40 min, and within-person autocorrelation of 0.3. The study has greater than 80% power to detect an immediate level change of 10% in daily active time at dashboard onset (18 min; *α* = 0.05, two-sided), and greater than 99% power for a 15% level change (27 min, within-person d = 1.08). The study is primarily powered for the immediate level change parameter (*γ*₂₀); detection of the sustained slope change parameter (*γ*₃₀) would require larger per-day effects given the relatively short post-onset observation period. The sample of 50 provides adequate headroom for anticipated data attrition (early discharge, camera downtime) and planned subgroup analyses by functional level. We note that, while the sample is adequately powered for the primary immediate level-change parameter, the planned functional-level subgroup comparisons and the sustained slope-change parameter are powered to detect only larger effects given the shorter post-onset observation window; these analyses are therefore pre-specified as exploratory and hypothesis-generating rather than confirmatory.

### Randomization and blinding

Randomization of dashboard onset timing will be performed using a computer-generated random number sequence, consistent with recommendations for randomized SCEDs (27, 28). Allocation will be concealed using sequentially numbered, opaque, sealed envelopes prepared by a statistician not involved in recruitment. Patients will be stratified by primary diagnosis (stroke vs. non-stroke) and baseline functional level [dependent [FIM motor score ≤58] vs. independent [FIM motor score >58]] to ensure balanced representation across onset strata. Neither patients nor clinical staff can be blinded to dashboard activation, as the intervention involves an active visual feedback display. However, the primary outcome (daily active time) is measured objectively and continuously by the PreSAGE system, eliminating observer bias in outcome ascertainment. The statistician conducting the primary analysis will be blinded to onset stratum assignments.

### Qualitative component: semi-structured interviews

A qualitative process evaluation will be conducted following Phase 3 data collection, consistent with the explanatory sequential design (34). The qualitative component follows the Medical Research Council framework for process evaluation of complex interventions (35) and will explore: (a) participant experiences with the monitoring system and dashboard, (b) perceived mechanisms of effect (or lack thereof), (c) barriers and facilitators to engagement, (d) privacy perceptions, and (e) contextual factors influencing implementation.

Purposive sampling will recruit participants representing diverse perspectives:

**Patients (*n*** **=** **10–15):** Sampled for maximum variation in age, diagnosis, functional level, and dashboard engagement level (high, moderate, and low users as defined by interaction frequency terciles). Interviews will be conducted within 48 h prior to planned discharge.
**Ward sisters (*n*** **=** **2–3):** Senior nursing staff responsible for resource planning and dashboard-informed workflow decisions.
**Clinicians (*n*** **=** **4–6):** Including therapists (physiotherapists and occupational therapists) and physicians involved in dashboard-informed goal setting and clinical decision-making.

Topic guides will be developed based on the study objectives, piloted with two participants from each group, and iteratively refined. Interviews will be audio-recorded with consent, transcribed verbatim, and de-identified. Analysis will use reflexive thematic analysis (43) following Braun and Clarke's six-phase approach: familiarization with the data, generating initial codes, constructing themes, reviewing themes, defining and naming themes, and writing up. Two researchers will independently code 25% of transcripts; discrepancies will be resolved through discussion to enhance analytical rigor. Thematic saturation will be assessed using an information power framework (44), considering the study's specific aims, sample specificity, and theoretical positioning. NVivo (Lumivero) will be used for data management.

### Mixed-Methods integration

Integration of quantitative and qualitative findings will follow a joint display approach (37). A convergence matrix will be constructed to systematically compare quantitative activity trajectories with qualitative themes across key dimensions: engagement mechanisms, barriers to use, differential response patterns, and contextual influences. Specifically, qualitative themes will be mapped to quantitative subgroup analyses (e.g., whether patients who describe high motivation in interviews also demonstrate steeper post-onset activity increases, and whether patients who report usability barriers show lower dashboard interaction rates and smaller activity gains). The meta-inference will address: does the DREAMT intervention improve activity levels, for whom, through what mechanisms, and under what conditions (34, 36).

### Data analysis

#### Quantitative analysis

The primary analysis will use a piecewise multilevel growth model (42, 45) to estimate the within-person effect of dashboard activation on daily active time. This analytic approach is well-established for combining data from multiple SCEDs into a unified multilevel framework (42). The two-level model (Level 1: daily observations nested within Level 2: patients) will take the form:

*Level 1: Y*ti* = β*0i* + β*1i*(Day*ti*) + β*2i*(Phase*ti*) + β*3i*(Day_post*ti*) + e*ti
*Level 2: β*0i* = γ*00* + u*0i*; β*1i* = γ*10* + u*1i*; β*2i* = γ*20* + u*2i*; β*3i* = γ*30* + u*3i

where Yti is the daily active time for patient *i* on day *t*; Dayti is the centered day variable; Phaseti is a binary indicator (0 = baseline, 1 = post-dashboard); Day_postti captures the post-activation trend (0 during baseline, days since activation during intervention); *β*2i captures the immediate level change (jump in activity at dashboard onset); *β*3i captures the change in slope (sustained trend change); and eti is the residual error modeled with an AR(1) structure to account for serial autocorrelation.

The Level 2 equations model between-patient variation in intercepts and slopes as random effects. Fixed effects (*γ*00, *γ*10, *γ*20, *γ*30) represent the average population-level baseline, pre-intervention trend, level change, and slope change. The primary hypothesis test is that *γ*20 (average immediate level change) and/or *γ*30 (average sustained slope change) differ significantly from zero.

Sensitivity analyses will include: (a) adjusting for patient-level covariates (age, diagnosis, baseline FIM, Clinical Frailty Scale score) in Level 2 equations; (b) testing different lag specifications (immediate vs. 1-day delayed effect); (c) modelling onset stratum as a fixed effect to test whether timing of activation moderates the intervention effect; (d) excluding patients with fewer than 14 days of total data (approximately 18% based on Phase 1 length of stay distribution); and (e) using Bayesian estimation as a robustness check. All analyses will follow the intention-to-treat principle based on assigned onset day.

Secondary analyses for clinical outcomes (FIM, FAC, MBI, length of stay, discharge destination, complications) will use generalized linear models adjusted for baseline characteristics. Dashboard utilization patterns will be explored using descriptive statistics and latent class analysis to identify distinct engagement profiles. Subgroup analyses will compare intervention effects between functionally dependent and independent patients, with the hypothesis that dependent patients may show primary improvements in clinician-mediated outcomes (nursing engagement frequency, time to first sit-out, complication rates) while independent patients may show primary improvements in self-directed activity outcomes (daily active time, exercise compliance, dashboard utilization). The dose-response relationship between dashboard interaction frequency and activity changes will be examined using the interaction count as a time-varying covariate in the multilevel model.

All quantitative analyses will be conducted using R (R Foundation for Statistical Computing, Vienna, Austria) with the “lme4”, “nlme”, 'SingleCaseES', and “brms” packages.

#### Qualitative analysis

Reflexive thematic analysis (43) will proceed through both inductive and deductive coding, with initial codes informed by the study objectives and the Theoretical Domains Framework (46) while remaining open to emergent themes. A codebook will be developed iteratively and refined through team discussion. Inter-coder reliability will be assessed on 25% of transcripts using Cohen kappa (*κ* ≥ 0.70 target). Member checking will be offered to consenting participants who wish to review a summary of findings.

#### Mixed-Methods analysis

Following the joint display framework (37), quantitative activity trajectories and qualitative themes will be presented in an integrated matrix organized by study objective. Specific attention will be given to explanatory connections: whether qualitative accounts of motivation, self-efficacy, or technology acceptance help explain observed quantitative variation in activity trajectories and dashboard engagement patterns. Cases of convergence (quantitative and qualitative findings align), divergence (findings conflict), and expansion (qualitative data elaborate or extend quantitative findings) will be explicitly identified and discussed (36).

## Ethical considerations

This protocol reports research involving human participants, including recruitment, continuous monitoring, and semi-structured interviews. Ethical approval has been obtained from the National Healthcare Group Domain-Specific Review Board (DSRB Reference: 2024-3489). Written informed consent will be obtained from all participants or their legally authorized representative. The thermography-based system captures heat signature data only, without personally identifiable visual information, preserving patient privacy. All data will be pseudonymized and stored on secure, encrypted servers compliant with Singapore's Personal Data Protection Act. Participants may withdraw at any time without affecting their clinical care.

For the qualitative component, separate written consent will be obtained for audio recording. Interview transcripts will be de-identified and stored separately from consent forms. A data safety monitoring plan has been developed, with quarterly independent review of adverse events. The study poses minimal risk to participants, as the intervention provides supplementary information to standard rehabilitation care without modifying clinical treatment protocols.

## Results

As of May 2026, the study is in Phase 1. Forty-six of 50 planned patients have been recruited, with one patient withdrawn, yielding 45 evaluable participants. The median rehabilitation length of stay among recruited patients is 24 days (mean 27.3, SD, 17.0, IQR, 15–35, range 4–70), with 81.8% having stays of 14 days or longer. Of these, 330 patient-days have been manually labelled to establish ground truth activity classifications, and 268 patient-days have been evaluated using the AI prediction model.

To improve ground truth data for nursing engagement detection, recruitment has been extended to Ward 15G, a general surgical ward equipped with Bluetooth tracking tags for nursing staff. Approximately 10 patients from Ward 15G are planned, enabling accurate capture of nurse-presence duration alongside thermographic data. This approach is expected to strengthen ground truth labelling for the nursing engagement algorithm. A new hospital data governance policy introduced on 1 April 2026 restricts vendor access to raw data from the PreSAGE server; data extraction is now conducted by designated hospital staff through approved Synapse accounts. This has temporarily slowed data transfer but does not affect data integrity or study continuity.

Preliminary evaluation of the patient state recognition algorithm demonstrates promising accuracy. For a representative participant (Patient 3), overall daily accuracy ranged from 90% to 97% across seven consecutive days. F1 scores for individual states were: 0.97 for lying on bed (precision 0.99, recall 0.95), 0.87 for sitting on bed edge (precision 0.78, recall 0.98), and 0.90 for out of bed/on chair (precision 0.90, recall 0.90). Across the majority of patients and days evaluated, F1 scores exceed 0.80 for all patient states.

Exercise detection has been categorized by movement magnitude: expansive exercises (e.g., shoulder flexion, hip abduction) are detected with reasonable accuracy; non-expansive exercises (e.g., core stabilization, heel slides) demonstrate limited detection capability; and small-motion exercises (e.g., fist clenching, ankle dorsiflexion) are not reliably detectable by the thermographic system. Assisted exercises are challenging to classify when the assisting person occludes the patient from the camera. Exercise detection is subject to a high false positive rate, with precision estimated at approximately 70% in current evaluation; this reflects the algorithm's tendency to classify repetitive gross movements (e.g., conversational hand gestures, positional adjustments) as exercise. Recall for true exercise episodes remains acceptable, but the low precision necessitates further model refinement prior to Phase 3 deployment.

The system occasionally misclassifies uncommon lying postures as sitting, an issue being addressed through model refinement with additional training data. Three patients remain in the ward at the time of this report. Phase 2 algorithm validation and dashboard co-design are expected to commence in mid-2026. The System Usability Scale instrument has been integrated into the data collection platform in preparation for Phase 2 usability testing. A dashboard co-design meeting was held in April 2026 with clinicians, nursing staff, and the technology partner (CoNEX Healthcare). Key design decisions reached include: (1) a patient activity timeline with hourly granularity using stacked colour bars distinguishing active and inactive periods; (2) a day/night toggle (06:00–22:00 daytime; 22:00–06:00 nighttime) for rest quality and nursing engagement review; (3) a minimum episode threshold of one minute for sitting-out-of-bed counts to reduce noise from transient postural changes; (4) a toggle differentiating assisted from unassisted bed exits to support nursing risk assessment; and (5) a ward-level overview dashboard displaying color-coded nursing engagement duration per bed to support shift-level resource allocation. Patient-facing reporting via printed daily summaries is planned as an interim implementation strategy pending tablet integration, with Phase 3 RMBD evaluation anticipated from end 2026. Final results are expected by 2027.

## Discussion

### Principal considerations

This protocol describes a rigorous mixed-methods approach to evaluating a novel thermography-based AI monitoring and feedback system in inpatient rehabilitation. The randomized multiple baseline design across participants represents a significant methodological advance over the pre-post and simple interrupted time series designs that predominate in digital health evaluations in rehabilitation settings.

The RMBD is well-suited to this study context for several reasons. First, the continuous within-patient time-series data generated by thermographic monitoring creates an unusually rich dataset that is best exploited by designs that model individual trajectories rather than group means at discrete timepoints. Second, the randomization of dashboard onset timing across participants provides a credible basis for causal inference: if activity increases systematically coincide with dashboard activation at five different randomly assigned timepoints, the effect is unlikely attributable to maturation, history, or regression to the mean (27, 28). Third, the design is ethical, as all patients receive the intervention during their admission; the only variation is the timing of onset. Fourth, the design does not require a separate no-treatment control group or multiple wards, making it feasible within a single-site context.

The piecewise multilevel growth model further strengthens the analysis by simultaneously modelling the natural recovery trajectory (pre-intervention slope), the immediate effect of dashboard activation (level change), and the sustained effect (post-intervention slope change), while accounting for between-patient heterogeneity and within-patient autocorrelation (42, 45). This provides more nuanced inference than a simple pre-post comparison.

### Comparison with prior work

To our knowledge, this is the first study to employ thermography-based continuous monitoring with AI-driven activity recognition combined with a patient-facing feedback dashboard in inpatient rehabilitation. Prior studies using wearable accelerometers (10–13) have characterized rehabilitation activity levels but have not closed the feedback loop with real-time patient-facing data. The RMBD also represents a novel application of SCED principles to digital health technology evaluation, extending the design tradition from its origins in behavioural and rehabilitation intervention research (27, 30, 33) to health informatics. Although the multiple baseline design is well-established in SCED methodology, its application with moderately sized samples (*n* = 50) analysed through multilevel modelling represents an innovative scaling of this approach that bridges traditional SCED methodology with group-level inference (42).

The mixed-methods design is aligned with the Medical Research Council framework for evaluating complex interventions (35), which emphasizes understanding not only whether an intervention works but also how and why, and what contextual factors shape implementation. The qualitative process evaluation will generate the mechanistic and contextual insights essential for refining the intervention and planning scale-up.

### Limitations

Several anticipated limitations should be acknowledged. First, the thermographic camera has a limited field of view covering only the bedside area, patient activities outside this zone (therapy gymnasium, bathroom visits, corridor ambulation) are not captured, potentially underestimating total daily activity. A check-in/check-out mechanism will partially mitigate this gap. Second, the single-site design may limit external generalizability, although the ICH serves a patient population typical of tertiary rehabilitation facilities in Singapore, and the qualitative component will explore transferability considerations. Third, the RMBD, while providing strong within-patient causal inference, relies on the assumption that the natural recovery trajectory is adequately modelled by the pre-intervention data points; patients with very short baseline periods (3 days) may have less stable baseline estimates (27, 41). Phase 1 data show substantial length of stay variability (SD 17.0, range 4–70 days), with 18.2% of patients staying fewer than 14 days; patients with short stays assigned to later onset strata will contribute limited post-onset data, though multilevel modelling accommodates unbalanced observations (27, 41). The inclusion of five onset strata and the multilevel modelling approach partially address this concern. Fourth, patients and staff cannot be blinded to dashboard activation, potentially introducing performance or Hawthorne effects; however, the objective, automated nature of the primary outcome measure minimizes this risk. Fifth, the system cannot detect small-motion exercises, and person identification within the camera's field of view remains a technical challenge when visitors or staff are present, although filtering algorithms are in development. Sixth, while the study substantially exceeds minimum SCED standards with 50 participants and five onset strata, the non-concurrent enrolment of participants (patients are recruited as they are admitted) means that the design functions as a non-concurrent MBD, which has been noted to have somewhat weaker internal validity than a fully concurrent design (31, 32); however, the sample size of 50 participants and randomization of onset timing substantially mitigate this concern. Seventh, the DREAMT intervention is multi-component, comprising continuous monitoring, a patient-facing dashboard, therapist-guided goal setting, and a clinician-facing dashboard. Because these elements are introduced together at dashboard activation, the design cannot isolate the independent contribution of any single component, and observed effects should be interpreted as those of the integrated intervention package; the qualitative process evaluation and the planned dose–response analyses of dashboard interaction will be used to generate, but not confirm, hypotheses about which components drive change. Finally, several stated advantages of the design and technology are anticipated rather than demonstrated, and the comparative and causal claims advanced in this protocol will require empirical confirmation once the study is complete.

## Conclusions

The DREAMT study represents a comprehensive evaluation of a privacy-preserving, AI-powered activity monitoring and feedback technology for inpatient rehabilitation. The randomized multiple baseline design across participants exploits continuous within-patient monitoring data to provide causal inference that is stronger than conventional pre-post or interrupted time series designs and more feasible than cluster randomized trials in a single-site context. The embedded qualitative process evaluation will illuminate the mechanisms and contextual factors that shape intervention effectiveness. If the DREAMT intervention proves effective, it could fundamentally transform rehabilitation monitoring from episodic, subjective observation to continuous, objective, personalized, data-driven care thereby optimizing both patient outcomes and nursing resource allocation.
